# The role of particulate guanylyl cyclase B (GC-B) in auditory function in adult mice

**DOI:** 10.1186/2050-6511-16-S1-A102

**Published:** 2015-09-02

**Authors:** Steffen Wolter, Dorit Möhrle, Dennis Zelle, Marlies Knipper, Hannes Schmidt, Lukas Rüttiger

**Affiliations:** 1 University of Tübingen, Department of Otolaryngology, Tübingen, Germany; 2 Max Delbrück Center for Molecular Medicine, Developmental Neurobiology, Berlin, Germany

## Background

cGMP signaling triggered by the binding of C-type natriuretic peptide (CNP) to its receptor guanylyl cyclase B (GC-B; NPR2; NPRB) has been linked by genetic evidence to a remarkable variety of physiological functions like skeletal bone growth, female fertility, cardiac growth, fat metabolism and gastrointestinal function. For the nervous system it has been recently demonstrated that the CNP/GC-B/cGMP/cGMP-dependent protein kinase type I (cGKI) signaling pathway is essential for sensory axon branching at the dorsal root entry zone of the spinal cord and at the rhombomeres of the hindbrain during embryonic development [1]. Also in the auditory system, distinct auditory nerve fiber (ANF) types that differ in their discharge rate and sound sensitivity bifurcate in the cochlear nucleus (CN), sending collaterals to the anteroventral, posteroventral, and dorsal subdivisions. The lack of GC-B has been shown to lead to a central phenotype [2].

## Results

Here, we describe that the lack of GC-B in addition leads to a peripheral phenotype which is manifested in auditory threshold loss and altered wave amplitudes and latencies of stimulus-evoked auditory brainstem responses (ABR). Our preliminary results indicate that this deficit is related to a combined afferent and efferent fiber phenotype.

## Outlook

We will further investigate the functional consequences of bifurcation loss of ANF on central hearing function and central plasticity. This may lead to sound localization problems, a progressive aging phenotype [3] and an increased risk for tinnitus [4].
